# Meta-analytic study of implant survival following sinus augmentation

**DOI:** 10.4317/medoral.16920

**Published:** 2011-12-06

**Authors:** Jessica Cabezas-Mojón, Cristina Barona-Dorado, Gerardo Gómez-Moreno, Fernando Fernández-Cáliz, José-María Martínez-González

**Affiliations:** 1 Dentist. Master in Buccal Surgery and Implantology, University Hospital, Madrid, Spain; 2 Associate Lecturer in Buccal Surgery, Faculty of Dentistry, Complutense University of Madrid, Spain; 3 Contract Lecturer in Special Patient Care, Faculty of Dentistry, University of Granada, Spain; 4 Associate Lecturer in Buccal Surgery, Faculty of Dentistry, Complutense University of Madrid. Lecturer on Master’s program in Buccal Surgery and Implantology, University Hospital, Madrid, Spain; 5 Senior Lecturer in Maxillofacial Surgery, Faculty of Dentistry, Complutense University of Madrid. Director of the University Hospital Buccal Surgery and Implantology Service, Madrid, Spain

## Abstract

Objectives: To evaluate graft types used for maxillary sinus augmentation and review success rates of dental implants inserted in these areas, analyzing the graft materials used, implant surface types and the moment of implant placement. Study Design: A meta-analytic study reviewing articles on sinus augmentation published during the last ten years. Results: 3,975 implants placed in sinus augmentations (with bony windows) were registered, of which 3,749 implants survived, a survival rate of 94.3%. Conclusions: When performing sinus augmentation, bone substitute materials are just as effective as autologous bone, whether used alone or in combination with autologous bone. Implant surface treatments can have an important effect on implant survival and it would appear that roughened surfaces are the best option. When implants are inserted simultaneously to grafting, a higher failure rate can be expected.

** Key words:** Sinus augmentation, bone implant, bone regeneration, dental implant.

## Introduction

When bone height/width are insufficient (according to some authors, minimum requirements are 10 mm and 1 mm of bone to each side of the implant respectively), implant placement is impossible or may be accompanied by serious esthetic defects following prosthodontic rehabilitation (1,2). 

Current concepts in implantology assert that implants must be placed according to the needs of a predetermined restoration rather than the bone volume available. As a result, the balance between function, biology and esthetics often require the restoration of alveolar bone in order to meet normal bone height and width parameters (2-5).

Furthermore, rehabilitation in edentulous regions may be complicated by crestal bone atrophy following tooth extraction/loss that may be or may be not associated with periodontal disease. Sinus pneumatization, together with poor bone quality, is one of the most challenging circumstances in implantology, a condition that will restrict implant placement in such areas (3,4).

When these situations occur, bone grafts can be used to correct the bone deficits, allowing the placement of implants of adequate length and width. There is a diverse choice of graft materials available for replacing bone lost through atrophy, trauma or congenital pathological processes. These graft materials include: intra or extraoral autologous bone, heterologous grafts, alloplastic grafts, xenografts or a combination of these (3).

For decades, researchers have sought graft materials with specific characteristics to respond to the needs of each reconstruction situation. This has involved the study and comparison of the benefits offered by potential bone donor sites in order to reduce potential risks and complications during graft integration or ‘take’ and improve treatment success rates (4). In this way, research has made a close observation of the behavior of each graft material:

 Autograft (autologous/autogenous): tissue or cell tissue from some other part of the patient’s body. This may include cortical, spongy cortical or spongy bone grafted in either block or particle form. Care must be taken to avoid disease transmission and immune rejection. With respect to graft structure, it must be said that cortical bone grafts have greater structural strength, greater osteo-conductive capacity and undergo lower resorption. However, they are poor in osteogenic cells. 

Spongy bone grafts, on the other hand, are rich in osteogenic cells and revascularization is faster. But this material has the disadvantage of a lack of rigidity and lower resistance to resorption. 

 Allograft: bone graft taken from one individual for implantation in another of the same species. The most usual material is lyophilized bone, whose most remarkable characteristic is its osteoconductivity. It is marketed in block form or as shavings but suffers high resorption once grafted. There are three types of bone allografts: frozen, lyophilized and demineralized. 

 Xenograft: Implant material obtained from an animal source (4-6).

Bone substitutes: Amongst the materials used for regeneration that do not contain a bone matrix, this is a heterogeneous group of materials that include hydroxyapatite and growth factors (5-7).

In general, the success of a bone graft is measured in terms of its capacity to withstand the conditions of tension and mechanical deformation to which it is subjected (8).

The interactions between graft material and healing processes at the host site have a direct influence on the pattern, rate and quality of new bone formation. Successful grafts are those that undergo revascularization and substitution of the graft material by host bone, without suffering a significant loss of mechanical strength or volume (2). Under radiological examination, bone grafts will be seen to lose their original shape (8).

## Material and Methods

A meta-analytic study was performed of articles sourced through a bibliography search using PubMed. Key search words were: sinus augmentation, bone implant, bone regeneration, and dental implants. Articles published between 1999 and 2010 were reviewed and 20 articles were selected, of which 16 were included for statistical analysis as they fulfilled inclusion criteria with the following parameters: 

• Graft type used.

• Survival rate. 

• Implant surface type (machined or roughened). 

• Residual bone height. Quantity of bone in millimeters existing prior to sinus lift. 

• Moment of implant placement (in a single phase, at the same moment as sinus augmentation, or as a second later surgical intervention). 

• Follow-up period (number of months following the placement of implant-supported prostheses). 

Data registered were entered in a working table ( Table 1) and statistical analysis of the results was performed.

## Results

A total number of 1,318 patients were registered with 3,975 implants placed into sites that underwent maxillary sinus augmentation. In all cases surgery was performed through bony windows filled with graft material. Implants were placed either as part of a single surgical operation, at the same moment as grafting, or as a second later intervention, depending on study protocol. 

Out of the total of 3,975 implants inserted, 3,749 survived, a survival rate of 94.3%. 

With regard to subject sex, out of 1,318 patients, 53% were women and 47% men. The average subject age was 51.3 years ranging between 18 and 80 years.

In an analysis of the articles reviewed, sinus augmentation was performed using autologous graft in 59% of patients, 24% received a combination of autologous bone and bone substitute, 10% were treated with bone substitute alone and in 7% of cases the graft material was not specified. 

In cases of autologous graft, 82% were harvested from intraoral donor sites such as the symphyseal area, the mandibular ramus, tuberosity or obtained as a result of bony window osteotomy. In the remaining 18%, bone was taken from extraoral donor sites such as the calota? cranium or the anterior or posterior iliac crest. 

With regard to the graft material used for sinus augmentation, a total of 2,514 implants were placed in autologous grafts with an overall survival rate of 2,334 implants, a survival rate of 93%. A total of 833 implants were placed in grafts combining autologous bone with bone substitutes and 804 survived, a survival rate of 96.5%. 345 implants were inserted in bone substitutes with 330 surviving, a survival rate of 95.6%. 

Out of the total number of implants inserted, 428 had smooth machined surfaces and showed a survival rate of 75.6%; 3.229 had roughened surfaces, with a survival rate of 96.8%. 

Initial bone height varied between 0 mm and over 9 mm. Some authors inserted implants whenever bone height exceeded 3 mm, while others delayed implant placement to a second intervention in spite of the availability of some 9 mm bone height. 

With regard to the moment of implant placement, 948 implants were placed at the same time as sinus augmentation with an overall survival rate of 900 implants, a success rate of 94%. 2,433 implants were inserted in a second later intervention, with the survival of 2,355 implants, a success rate of 96.8%. Of these implants placed as a later surgical procedure, 1,787 were inserted between four and six months following sinus augmentation and the rest (646) between six and ten months. 

The time between implant insertion and prosthesis placement varied between four and six months in 2,154 cases. A further 919 implants were loaded at the six-month mark, 433 implants between 6 and 12 months and lastly 135 implants were loaded between 16 and 18 months following insertion.

Follow-up periods in the articles reviewed, counted from the time of prosthesis attachment/loading, ranged between six months and twelve years.

## Discussion

The indications for sinus augmentation have multiplied, together with the predictability of implant treatment outcomes, and thanks to sinus lift procedures it is now possible to place implants of adequate length in posterior maxillary areas (4,9).

From the start, autologous bone grafting has been the main technique used for sinus augmentation. Autologous bone is considered the ‘gold standard’ for intraoral implantation due to its osteoconductive and osteoinductive properties. However, there are some inconvenient aspects to the procedure, including the possible need for hospitalization during extraoral bone-harvesting procedures or the need for a second intraoral donor site, which will lead to increased morbidity. Some research articles state that autologous bone resorbs at an above-average rate, which can lead to posterior pneumatization of the sinus and/or implant failure (10-17).

The maximum failure rate observed in research has been with autogenous grafting. A series of influential factors have been put forward to explain this failure rate including: inadequate graft material volume, the influence of osseous coagulate, simultaneous implant placement before adequate healing has taken place and the implant surface chosen for insertion at these sites (5,9).

In this type of sinus augmentation surgery, the most frequent complication is perforation of the sinus membrane, which occurs in some 16.7% - 44% of cases. The articles reviewed describe the consequences as possible post-operatory inflammation and an increased implant failure rate, which may reach 30%. In such cases the repair of the membrane will prolong and complicate surgery (10,15).

Furthermore, the use of autologous bone, whether harvested intraorally or extraorally, involves an increase in morbidity, which patients may find unacceptable. 

The limitations of autogenous grafts can be overcome by using bone substitutes either completely replacing or minimizing the use of autologous bone. As described above, numerous allogenic or alloplastic materials have been developed, which may be used alone or in combination with autogenous bone. The articles reviewed show that these materials can be as effective as autologous bone (2,4,5,7,9-12,14,15,17,18-20). They also found a higher survival rate in cases in which autogenous bone was grafted as particles than in block form. 

Histologic evidence generated by studies of mature grafts and the excellent survival rates of implants inserted in them have led to the realization that these non-autogenous graft materials may be considered an excellent option (4-6,9,10,13,16,18,20). 

Cordaro et al. (1), in a study using block grafts harvested from the jaw and used for 3D reconstruction, observed an average lateral increase of 5.5 mm, shrinking to 4.3 (p< 0,01) during the healing process; the average vertical increase was of 3.2 mm, reducing to 2.1 mm (p<0,01). 

In a study of non-autogenous graft materials, Frenken et al. (5) evaluated the quantity and quality of bone formed in sinus augmentations using a synthetic material: biphasic calcium phosphate consisting of a combination of 60% hydroxyapatite and 40% ß-tricalcium phosphate. Their histologic study observed direct contact between bone and bone substitute; new mineralization tissue formation was observed in the cranial region of the original alveolar bone. 

Implant surface may also influence implant survival rates as surface texture can help coagulate to cling directly to the implant surface, while it will slide from a smooth surface (4).

Given the importance of primary stability for implant integration, the design of both microstructures and macrostructures has a special importance as it can help to achieve primary stability even in situations where bone volume is scant. Rough surfaces produce contact osteogenesis, this is to say, bone apposition taking place on the implant surface. This process leads to a more favorable bone-to-implant interface than the distance osteogenesis that occurs with smooth implant surfaces, when bone formation takes place close to the implant but not in direct contact with it (3,4,9,12).

With regard to the time of implant placement, in some of the articles reviewed implant survival rates were similar independently of whether treatment protocols opted for immediate implant insertion or delayed insertion (4). However, other articles make it clear that most failures are produced in implants inserted simultaneously to the graft procedure, regardless of the graft material used or the type of implant surface. A high frequency of failure was seen to be caused by poor primary stability, premature non-functional loading resulting from mastication or low bone density (4,6,7,9,12,13,15-17).

The choice of technique is often determined by the quantity of residual crestal bone available; if there is not sufficient bone volume to guarantee primary stability, delaying implant insertion until the graft has matured is a necessity. A multi-step approach can overcome the deficiencies of markedly atrophied crestal bone, given that implants can be placed successfully in sites at which, prior to grafting, crestal bone height was 4 mm or less. (6,7,9-12,16)

If the graft, once it has healed adequately, is the main factor responsible for the implants’ mechanical and biological stability, the use of a composite graft material containing some autogenous bone should be considered. The increased volume of mineralized bone that results from such grafts can lead to greater graft stability, with greater bone-to-implant contact, and so offers better chances of implant survival. (4,9)

Some of the articles reviewed, such as those by Del Fabbro et al. (4) and Wallace et al. (11) have found sufficient evidence to support the use of membranes to cover sinus augmentation lateral windows; these studies found that vital bone formation was on average double in sinuses covered by membranes than uncovered and led to a higher implant survival rate. Bone formation is facilitated by the exclusion of the periosteum in the sinus graft’s regeneration process. Once lifted and repositioned, the periosteum loses its osteogenic potential and becomes fibrogenous, which might explain the process of invagination observed when a membrane covering is not used (4,11).

**Table 1:**
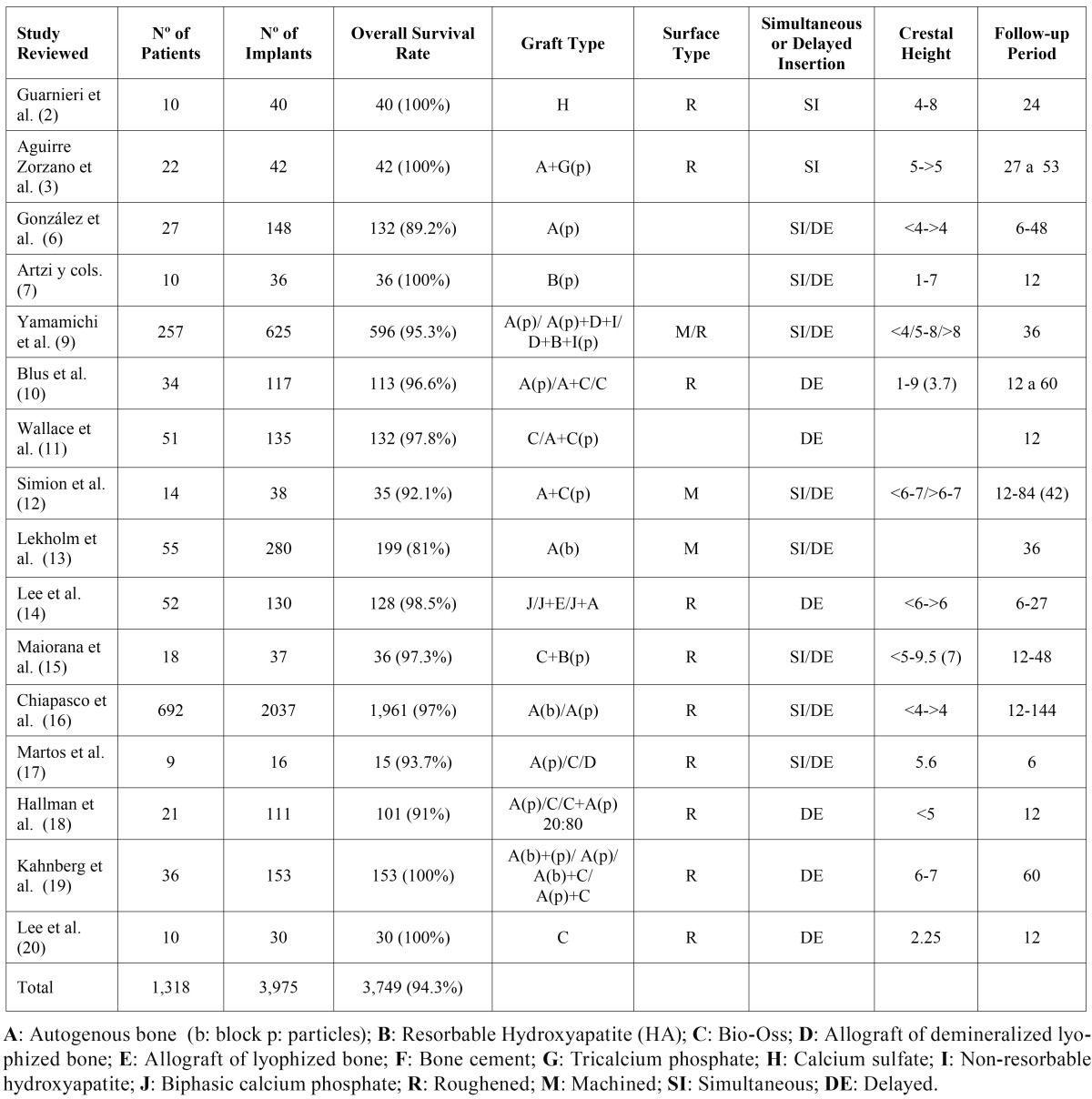
Working table showing all data.

## References

[1] Cordaro L, Torsello F, Accorsi Ribeiro C, Liberatore M, Mirisola di Torresanto V (2010). Inlay-onlay grafting for three-dimensional reconstruction of the posterior atrophic maxilla with mandibular bone. Int J Oral Maxillofac Surg.

[2] Guarnieri R, Grassi R, Ripari M, Pecora G (2006). Maxillary sinus augmentation using granular calcium sulfate (surgiplaster sinus): radiographic and histologic study at 2 years. Int J Periodontics Restorative Dent.

[3] Aguirre Zorzano LA, Rodríguez Tojo MJ, Aguirre Urizar JM (2007). Maxillary sinus lift with intraoral autologous bone and B--tricalcium phosphate: histological and histomorphometric clinical study. Med Oral Patol Oral Cir Bucal.

[4] Del Fabbro M, Testori T, Francetti L, Weinstein R (2004). Systematic review of survival rates for implants placed in the grafted maxillary sinus. Int J Periodontics Restorative Dent.

[5] Frenken JW, Bouwman WF, Bravenboer N, Zijderveld SA, Schulten EA, Bruggenkate CM (2010). The use of Straumann Bone Ceramic in a maxillary sinus floor elevation procedure: a clinical, radiological, histological and histomorphometric evaluation with a 6-month healing period. Clin Oral Implants Res.

[6] González-García R, Naval-Gías L, Muñoz-Guerra MF, Sastre-Pérez J, Rodríguez-Campo FJ, Gil-Díez-Usandizaga JL (2005). Preprosthetic and implantological surgery in patients with severe maxillary atrophy. Med Oral Patol Oral Cir Bucal.

[7] Artzi Z, Nemcovsky CE, Dayan D (2003). Nonceramic hydroxyapatite bone derivative in sinus augmentation procedures: clinical and histomorphometric observations in 10 consecutive cases. Int J Periodontics Restorative Dent.

[8] Bianchi AE, Vinci R, Torti S, Sanfilippo F (2004). Atrophic mandible reconstruction using calvarial bone grafts and implant-supported overdentures: radiographic assessment of autograft healing and adaptation. Int J Periodontics Restorative Dent.

[9] Yamamichi N, Itose T, Neiva R, Wang HL (2008). Long-term evaluation of implant survival in augmented sinuses: a case series. Int J Periodontics Restorative Dent.

[10] Blus C, Szmukler-Moncler S, Salama M, Salama H, Garber D (2008). Sinus bone grafting procedures using ultrasonic bone surgery: 5-year experience. Int J Periodontics Restorative Dent.

[11] Wallace SS, Froum SJ, Cho SC, Elian N, Monteiro D, Kim BS (2005). Sinus augmentation utilizing anorganic bovine bone (Bio-Oss) with absorbable and nonabsorbable membranes placed over the lateral window: histomorphometric and clinical analyses. Int J Periodontics Restorative Dent.

[12] Simion M, Fontana F, Rasperini G, Maiorana C (2004). Long-term evaluation of osseointegrated implants placed in sites augmented with sinus floor elevation associated with vertical ridge augmentation: a retrospective study of 38 consecutive implants with 1- to 7-year follow-up. Int J Periodontics Restorative Dent.

[13] Lekholm U, Wannfors K, Isaksson S, Adielsson B (1999). Oral implants in combination with bone grafts A 3-year retrospective multicenter study using the Brånemark implant system. Int J Oral Maxillofac Surg.

[14] Lee JH, Jung UW, Kim CS, Choi SH, Cho KS (2008). Histologic and clinical evaluation for maxillary sinus augmentation using macroporous biphasic calcium phosphate in human. Clin Oral Implants Res.

[15] Maiorana C, Sigurtà D, Mirandola A, Garlini G, Santoro F (2006). Sinus elevation with alloplasts or xenogenic materials and implants: an up-to-4-year clinical and radiologic follow-up. Int J Oral Maxillofac Implants.

[16] Chiapasco M, Zaniboni M, Rimondini L (2008). Dental implants placed in grafted maxillary sinuses: a retrospective analysis of clinical outcome according to the initial clinical situation and a proposal of defect classification. Clin Oral Implants Res.

[17] Martos Díaz P, Naval Gías L, Sastre Pérez J, González García R, Bances del Castillo F, Mancha de la Plata M (2007). Sinus elevation by in situ utilization of bone scrapers: technique and results. Med Oral Patol Oral Cir Bucal.

[18] Hallman M, Sennerby L, Lundgren S (2002). A clinical and histologic evaluation of implant integration in the posterior maxilla after sinus floor augmentation with autogenous bone, bovine hydroxyapatite, or a 20:80 mixture. Int J Oral Maxillofac Implants.

[19] Kahnberg KE, Vannas-Löfqvist L (2008). Sinus lift procedure using a 2-stage surgical technique: I Clinical and radiographic report up to 5 years. Int J Oral Maxillofac Implants.

[20] Lee YM, Shin SY, Kim JY, Kye SB, Ku Y, Rhyu IC (2006). Bone reaction to bovine hydroxyapatite for maxillary sinus floor augmentation: histologic results in humans. Int J Periodontics Restorative Dent.

